# Short hairpin RNA targeting Notch2 inhibits U87 human glioma cell proliferation by inducing cell cycle arrest and apoptosis *in vitro* and *in vivo*

**DOI:** 10.3892/mmr.2014.2661

**Published:** 2014-10-15

**Authors:** XUEZHEN LI, XIN HE, WEI TIAN, JIANZHEN WANG

**Affiliations:** 1 Department of Neurosurgery, Beijing Tiantan Hospital, Capital Medical University, Beijing 100050, P.R. China; 2 Department of Neurosurgery, Beijing Sanbo Brain Hospital, Capital Medical University, Beijing 100093;, P.R. China; 3 Department of Neurosurgery, Handan Central Hospital, Handan 056001, P.R. China; 4 Department of Neurosurgery, General Hospital of Chinese People’s Armed Police Forces, Beijing 100039, P.R. China

**Keywords:** Notch2, short hairpin RNA, cell cycle arrest, cell proliferation, apoptosis, glioma

## Abstract

Notch signaling has been reported to be oncogenic or tumor suppressive, depending on the tissue context. To investigate the effects of Notch2 knockdown on U87 human glioma cell proliferation *in vitro* and *in vivo*, and the associated mechanisms, U87 cells were stably transfected with p green fluorescent protein (GFP)-V-RS Notch2 short hairpin (sh) RNA plasmid and pGFP-V-RS scramble-shRNA plasmid. The former was referred to as the Notch2-shRNA group and the latter as the negative-shRNA group. mRNA and protein expression, cell proliferation, cell cycle and apoptosis were measured by reverse transcription-polymerase chain reaction, western blot analysis, 3-(4,5-dimethylthiazol-2-yl)-2,5-diphenyltetrazolium bromide analysis and flow cytometry using propidium iodide, respectively. Tumor volume, tumor weight and cumulative survival rate were determined in a nude mouse xenograft tumor model. Notch2 mRNA and protein expression in the Notch2-shRNA group were reduced by 87.6 and 94.5% compared with the negative-shRNA group (P<0.001). Notch2 knockdown significantly inhibited U87 cell proliferation after three days of culture (P<0.05). Notch2 silencing induced cell cycle arrest at G_0_/G_1_ phase by upregulation of p21 protein expression and downregulation of mini chromosome maintenance complex 2 and cyclin-D1 protein expression. Furthermore, knockdown of Notch2 also induced U87 cell apoptosis. On day 50 after inoculation, tumor weight in the Notch2-shRNA group was significantly lower than that in the negative-shRNA group (0.55±0.10 vs. 1.23±0.52 g; P<0.01). The cumulative survival rate was significantly longer in the Notch2-shRNA group compared with the negative-shRNA group (log rank test P=0.01). In conclusion, Notch2 silencing inhibited U87 glioma cell proliferation by inducing cell cycle arrest and apoptosis *in vitro* and *in vivo*. Thus, Notch2 may be a key therapeutic target for the treatment of glioma.

## Introduction

Glioma, the most common malignant tumor of the central nervous system, accounts for 30–50% of intracranial tumors. It is characterized by high cell proliferation rates and invasiveness. Treatment of glioma includes surgery, radiation and chemotherapy. However, the median survival rate of glioma patients is only 9–15 months (1–5). Furthermore, the therapeutic effects of such treatments are limited, particularly for high grade gliomas, where the incidence and mortality rate remain high with a post-operative median survival time of less than one year (6). Difficulty in completely removing tumors and the recurrence of cancer after treatment remain a significant barrier to long-term survival. To overcome these challenges, focus on the development of novel therapies for the treatment of glioma is required.

Novel treatments include targeting molecular pathways specifically activated within glioma cells. Studies have shown that glioma is in part caused by deregulation of the Wnt signaling pathway and this deregulation is crucial in regulating processes, such as the initiation, proliferation and development of glioma cells (7–9). Inhibition of activated abnormal tumor signaling pathways may be an effective therapy with which to kill tumor cells, suppress cell proliferation and induce cellular differentiation.

One such signaling pathway involved in the development of glioma is the Notch signaling pathway (10). This is activated following binding of the epidermal growth factor (EGF) repeat region in Notch receptors (Notch 1–4) to their ligands, δ-like-1, 3 and 4, and Jagged 1 and 2, which are commonly expressed on adjacent cells (11). Notch receptors are important members of a family of proteins involved in the growth and development of vertebrates and invertebrates, and are involved in cell proliferation and differentiation (11). Following binding, two enzymatic cleavages occur to release the Notch intracellular domain (NICD) from the plasma membrane, thus translocating the NICD into the cell nucleus, allowing this domain to perform its regulatory role (12,13).

Studies have reported that the Notch signaling pathway is involved in the initiation and development of tumors (14–21). The role of Notch signaling as an oncogenic or tumor suppressor varies with tumor type (22–27). Notch signaling can promote cell proliferation in acute T-cell leukemia, breast cancer, renal epithelial urothelial cancer and pancreatic cancer (16,18,19,21,28,29). By contrast, Notch signaling can induce cell cycle arrest in small cell lung cancer (19,30). Activation of different Notch1 receptors inhibits tumor growth, while activation of Notch2 receptors promotes tumor growth, leading to different effects on embryonic brain tumor cell proliferation (31). Notch receptors are important in glioma progression. It has been reported that the Notch1 receptor enhanced the proliferation of glioma cells (12). However, whether the Notch2 receptor also promotes cell proliferation remains unknown (31,32).

Studies by Chen *et al* (10,32), Reichrath *et al* (33) and Sivasankaran *et al* (30) have shown that substantial levels of Notch2 mRNA and Notch2 protein are detected in U87 human brain glioma cells. However, it has been reported that Notch2 expression varies in different glioma cells, such as astrocytoma and medulloblastoma cells (10,32–34). Xu *et al* (35,36) investigated the role of Notch2 in the U251 glioblastoma astrocytic cell line and found low expression of Notch2 (35,36). Consequently, it is clear that Notch2 expression levels vary depending on the type of glioma.

The aim of the current study was to determine the role of Notch2 in human glioma cell proliferation in an attempt to provide a direction for the development of a novel molecular therapy. To investigate the role of Notch2 in glioma cell proliferation, the U87 cell line was used. This is a primary human glioblastoma cell line with epithelial morphology, which was originally obtained from a 44-year-old patient with stage IV disease. Notch2 expression was downregulated in the U87 human glioma cells using the RNA interference method. Mini chromosome maintenance complex (MCM)2, p21 and cyclin-D1 are involved in the cell cycle, however, the impact of the Notch receptors remains unclear. Therefore, cell proliferation, cell cycle distribution, cell cycle-related proteins and cell apoptosis of U87 cells *in vitro* and *in vivo,* prior to and after RNA interference, were investigated.

## Materials and methods

### Cell culture

The U87 human glioblastoma cell line was obtained from the Shanghai Cell Bank of the Chinese Academy of Medical Science (Shanghai, China). The U87 cells were cultured with Dulbecco’s modified Eagle’s medium (DMEM; Gibco Inc., Billings, MT, USA) containing 10% fetal bovine serum (Invitrogen, Carlsbad, CA, USA), 100 U/ml penicillin and 100 U/ml streptomycin (Beyotime, Shanghai, China) in an incubator containing 5% CO_2_ at 37°C.

### Animals

Thirty specific pathogen free BALB/c female nude mice (age, 6 weeks; body weight, 20.0±2.5g) were purchased from (Beijing HFK Bioscience Co., Ltd., Beijing, China). Mice were housed at 20–25°C with 50±5% humidity, access to food and water *ad libitum* and a 12:12h light/dark cycle. Experiments were approved by the Medical Ethics Committee of the Second Affiliated Hospital of Hebei Medical University (Shijiazhuang, China). All procedures involving mice conformed to the Guide for the Care and Use of Laboratory Animals published by the National Institutes of Health (NIH Publication No. 85–23, revised 1996).

### Construction and identification of U87 cells stably transfected with plasmids

The p green fluorescent protein (GFP)-V-RS Notch2 short hairpin RNA (shRNA) plasmid was purchased from Beijing OriGene Technologies Co., Ltd. (Beijing, China). In this study, three treatments were designed. The U87 cells with no treatment were considered as a blank control, termed the nontransfection group. The plasmid pGFP-V-RS Notch2-shRNA containing Notch2-specific shRNA and the plasmid pGFP-V-RS negative-shRNA containing unspecific shRNA (Beijing OriGene Technologies Co., Ltd.) were considered as the Notch2-shRNA and negative-shRNA groups, respectively. These plasmids were transfected into U87 cells. Briefly, U87 cells were inocculated into 4-well plates (a density of 1×10^5^ cells/ml, 150 μl/well) and incubated overnight. The pGFP-V-RS Notch2-shRNA plasmid or pGFP-V-RS negative-shRNA plasmid, together with Lipofectamine 2000 and optimem (both Invitrogen; 1:2.5:250) were incubated for 20 min at room temperature (RT), forming a DNA-liposome complex. The complex (100 μl) was added to the 24-well plate after the culture media was removed and mixed evenly. The U87 cells were incubated in the media containing the complex for 6 h. After the supernatant was discarded, DMEM was added to the plate. Cells were incubated in the media containing the complex and DMEM for 24 h until they were ready to be passaged at a ratio of 1:10. The transfected cells were passaged into a vessel containing growth media of 1 μg/ml puromycin (Corning Inc., New York, NY, USA) and incubated until clonal cells of U87 were present. Cell clones were selected and inoculated onto a 96-well plate for incubation. During the incubation, puromycin was maintained at 1 μg/ml. When cells achieved 70% confluence, stably transfected cells were transferred to incubation flasks and analyzed by a CKX31-A11RC fluorescence microscope (OLYMPUS, Tokyo, Japan) for visualization of the green fluorescent protein included in the plasmid vector.

### Reverse transcription-polymerase chain reaction (RT-PCR)

Total RNA in stably transfected cells was extracted with the TRIzol (Invitrogen) method. RNA purity was determined using absorbance at 260 and 280 nm (A260/280) using a Nanodrop spectrophotometer (ND-2000; Thermo Scientific, Pittsburgh, PA, USA), and the integrity of the RNA was verified by electrophoresis on formaldehyde gels. The first cDNA sequence was synthesized according to the manufacturer’s instructions (Invitrogen). This cDNA sequence was used as a template for PCR amplification. Primer sequences were as follows: Forward: 5′-CCC AAT GGG CAA GAA GTC TA-3′ and reverse: 5′-CAC AAT GTG GTG GTG GGA TA-3′ for Notch2; and forward: 5′-CCA CCC ATG GCA AAT TCC ATG GCA-3′ and reverse 5′-TCT AGA CGG CAG GTC AGG TCC AC-3′ for the glyceraldehyde 3-phosphate dehydrogenase (GAPDH) control. All reactions involved initial denaturation at 94°C for 15 min followed by 30 cycles of 94°C for 60 sec, 58°C for 60 sec and 72°C for 60 sec. PCR products were separated on 1.5% agarose gel electrophoresis, examined under UV light and photographed by a UV transilluminator (Imagemaster, Pharmacia Biotech, Piscataway, NJ, USA). The mean gray value of each group was determined by Image J software (National Institutes of Health, Bethesda, MD, USA). Relative mRNA expression was normalized to GAPDH expression.

### Western blot analysis

Stably transfected cells were lysed in lysis buffer (Nanjing KeyGen Biotech., Co., Ltd., Nanjing, China) on ice for 10 min and centrifuged at 20,000 × g at 4°C for 10 min. Proteins were quantified using a Bicinchoninic Acid Protein Assay kit (Beyotime, Nantong, China). A total of 40 μg protein was loaded for 15% sodium lauryl sulfate-polyacrylamide gel electrophoresis. Proteins were transferred onto polyvinylidene fluoride fiber membrane (Millipore, Billerica, MA, USA), blocked with 5% non-fat milk powder in Tris-buffered saline with 0.05% Tween-20 (TBST) for 2 h at RT and incubated with the following primary antibodies at 4°C overnight: Rabbit polyclonal antibodies against Notch2, cyclin-D1, p21 and MCM2; and mouse polyclonal antibody against β-actin (Santa Cruz Biotechnology Inc., Santa Cruz, CA, USA). Membranes were rinsed in TBST three times, and incubated with the respective secondary antibodies for 1 h at RT (horseradish peroxidase-conjugated monoclonal goat anti-rabbit IgG and monoclonal goat anti-mouse IgG; Santa Cruz Biotechnology Inc.). Membranes were rinsed in TBST again and protein expression was detected with 3,3′-diaminobenzidine (DAB; Beyotime, Jiangsu, China). The mean gray value of each group was determined by Image J software. Relative mRNA expression was normalized to β-actin.

### Detection of cell proliferation with a 3-(4,5-dimethylthiazol-2-yl)-2,5-diphenyltetrazolium bromide (MTT) assay

Stably transfected cells were inoculated onto a 96-well plate at a density of 3,000 cells/well. Cell proliferation was determined by an MTT assay each day for one week. In brief, 5 mg/ml MTT (Nanjing KeyGen Biotech., Co., Ltd.; 20 μl) was added to each well, incubated for 4 h and then centrifuged at 3,000 × g for 5 min. The supernatant was discarded and 150 μl dimethyl sulfoxide was added to each well. The plate was incubated for 10 min on a shaker (Bühler GmbH, Braunschweig, Germany). The absorbance at 490 nm was determined using an automatic Enzyme Labeling instrument (Beijing Putian Instrument Ltd., Beijing, China). A cell proliferation curve was generated.

### Cell cycle distribution and apoptosis

Stably transfected cells were collected and fixed in precooled 70% ethanol overnight at 4°C. After washing with phosphate-buffered saline (PBS), RNAase A (125 U/ml; Molecular Probes, Eugene, OR, USA) and propidium iodide (50 μg/ml; Molecular Probes) were added, and cells were incubated in the dark at 4°C for 30 min. Cells were analyzed using a FACSCalibur flow cytometer (BD Biosciences, Franklin Lakes, NJ, USA), and cell cycle distribution (in the G_0_/G_1_, S and G_2_/M phases) was calculated using the ModFit LT 3.2 software (BD Biosciences). Sub-G_1_ was taken to represent apoptosis.

### U87 human glioblastoma mouse xenograft tumor model

Stably transfected U87 cells at logarithmic growth phase were collected and washed with serum free DMEM. Cell concentration was adjusted to 5×10^6^ cells/ml. Thirty nude mice were randomly assigned to the three groups with ten mice in each group. Under a sterile hood, mice were sterilized with 75% ethanol and injected with 0.1 ml U87 cells (the nontransfection group), U87 cells stably transfected with Notch2-shRNA (the Notch2-shRNA group) or scramble-shRNA (the negative-shRNA group) into the back of the neck. The diameter of the tumor in the greatest axis and shortest axis was measured with a vernier caliper every five days. The tumor volume was calculated according to the formula: Tumor volume (mm^3^) = DGD × DSD^2^ × 0.5. The growth curve of the tumor was drafted as a plot of tumor volume against inoculation.

### Immunohistochemistry (IHC) analysis of mouse tumor specimens

On day 40 after inoculation, four mice were selected from each group and sacrificed by cervical dislocation. The specimens were observed, weighed, fixed in 10% buffered formalin for 8 h and embedded in wax. Sections (3 μm-thick) from tumor tissue samples, were mounted on glass slides precoated with 3-aminopropyltriethoxysilane and dried for the IHC analysis (IHC kit, Mai Bio Co., Ltd., Shanghai, China) of Notch2 protein. Antibodies against Notch2 for IHC were the same as those used for western blot analysis. Integrated Optical Density was determined by ImagePro Plus 6 software (Media Cybernetics, Rockville, MD, USA).

### Cumulative survival rate of nude mice

Of the ten mice in each group, four mice were selected from each group for IHC, and the remaining six mice continued to be housed under standard conditions. Each day, tumor growth was examined until the mice succumbed to the tumors. Kaplan-Meier survival plots were used to determine the cumulative survival rate of nude mice for each group. Tumor weight was measured 50 days after the inoculation as pre-experiments revealed that significant differences in tumor weight appeared at this time period following inoculation.

### Statistical analysis

All data are expressed as the mean ± standard deviation and analyzed with an SPSS 13.0 software package (SPSS Inc., Chicago, IL, USA). Statistical significance was evaluated by one-way analysis of variance with the least significance difference test for post hoc analysis. Kaplan-Meier survival plots were generated, and comparisons between survival curves were made with the log-rank statistics. P<0.05 was considered to indicate a statistically significant difference.

## Results

### Notch2 mRNA and protein expression was stably and effectively downregulated by shRNA in U87 glioma cells

**Figure 1:**
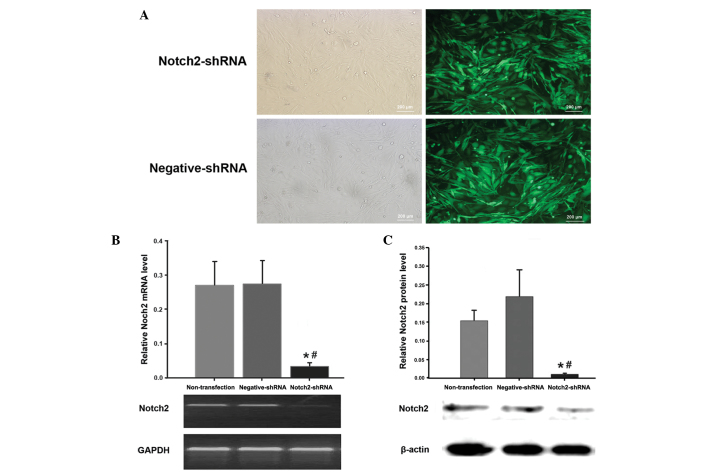
Knockdown of Notch2 by shRNA in glioma U87 cells. U87 cells were stably transfected with pGFP-V-RS Notch2-shRNA plasmid. (A) Monitoring transfection efficiency by GFP (magnification, ×200). (B) mRNA expression determined by RT-PCR with GAPDH as an internal control. (C) Protein expression determined by western blot analysis, normalized to β-actin. Data are presented as the mean ± standard deviation. ^*^P<0.05, compared with the nontransfection group; ^#^P<0.05, compared with the negative-shRNA group. shRNA, short hairpin RNA; RT-PCR, reverse transcription-polymerase chain reaction; GFP, green fluorescent protein; GAPDH, glyceraldehyde 3-phosphate dehydrogenase.

Transfection efficiency as monitored by GFP is shown in Fig. 1A. Notch2 mRNA and protein expression in U87 cells was determined by RT-PCR and western blot analysis, respectively. Compared with the negative-shRNA group, Notch2 mRNA (Fig. 1B) and protein (Fig. 1C) expression in the Notch2-shRNA group were reduced by 87.6 and 94.5%, respectively (P<0.05). In the nontransfection group and the negative-shRNA group, U87 cells showed no significant changes in the levels of Notch2 mRNA or protein (P>0.05).

### Effects of Notch2 knockdown on cell proliferation, cell cycle distribution and cell apoptosis in U87 cells (Fig. 2)

**Figure 2:**
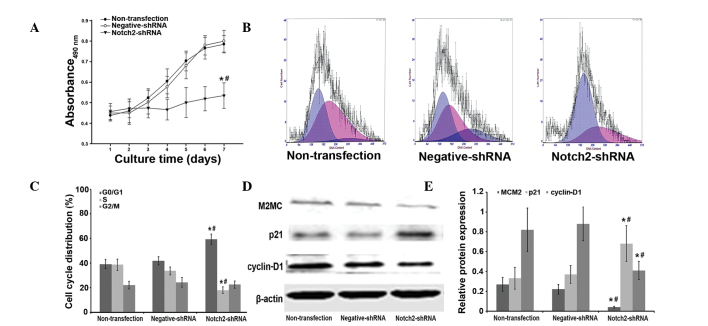
Effects of Notch2 knockdown on cell proliferation, cell cycle distribution and cell cycle-related protein expression in U87 cells. (A) Cell proliferation was determined by the 3-(4,5-dimethylthiazol-2-yl)-2,5-diphenyltetrazolium bromide assay. (B) Cell cycle distribution was analyzed by flow cytometry using propidium iodine staining. (C) Cell cycle distribution at the G_0_/G_1_, S and G_2_/M phases. (D) Expression of MCM2, cyclin-D1 and p21 protein were determined by western blot analysis. (E) Relative protein expression was normalized to β-actin. Data are presented as the mean ± standard deviation. ^*^P<0.05, compared with the nontransfection group; ^#^P<0.05, compared with the negative-shRNA group. shRNA, short hairpin RNA; MCM2, mini chromosome maintenance complex 2.

Compared with the nontransfection group and the negative-shRNA group, Notch2 knockdown significantly inhibited U87 cell proliferation after three days of culture (P<0.05). The highest inhibition rate was ~33.3% on day seven of culture (Fig. 2A). No significant difference was identified in the cell proliferation between the nontransfection group and the negative-shRNA group (all P>0.05).

Compared with the negative-shRNA group, the proportion of cells in S phase in the Notch2-shRNA group was significantly lower (18.1±2.7 vs. 33.7±3.3%; P<0.05), whilst the proportion in G_0_/G_1_ phase was significantly higher (59.4±4.1 vs 41.9±3.3%; P<0.05). The proportion of cells in the G_2_/M phase was not significantly different between the Notch2-shRNA group and negative-shRNA group (P>0.05; Fig. 2B and C). There was no significant difference in the cell cycle distribution between the nontransfection group and the negative-shRNA group (P>0.05).

**Figure 3:**
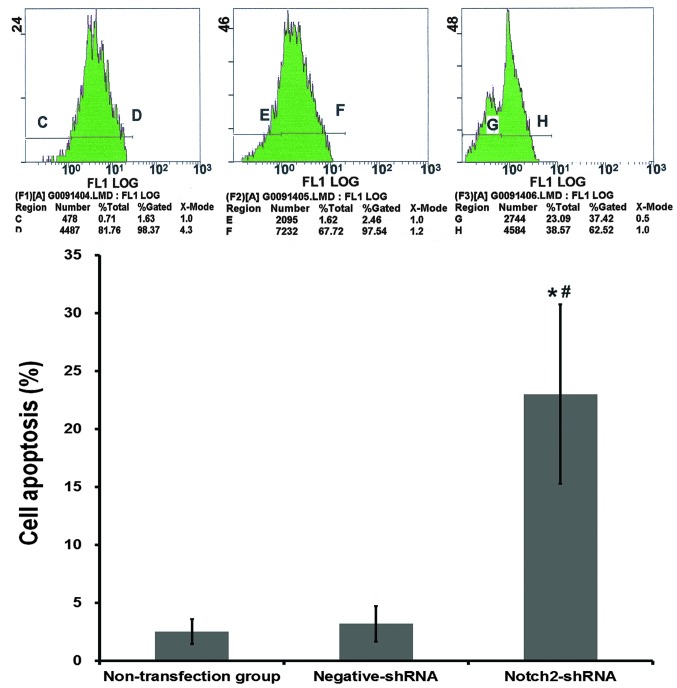
Effect of Notch2 knockdown on cell apoptosis in U87 cells. Sub-G_1_ phase represented cell apoptosis and was analyzed by flow cytometry using propidium iodine staining. Data are presented as the mean ± standard deviation. ^*^P<0.05, compared with the nontransfection group; ^#^P<0.05, compared with the negative-shRNA group. shRNA, short hairpin RNA.

Compared with the negative-shRNA group, the percentage of cells that had undergone apoptosis in the Notch2-shRNA group was significantly higher (23.00±7.74 vs. 3.21±1.53%; P<0.001; Fig. 3). There was no significantly difference in the percentage of cells that had undergone apoptosis between the nontransfection group and the negative-shRNA group (P>0.05).

### Effects of Notch2 knockdown on cell cycle-related protein expression

Western blot analysis showed that Notch2 silencing significantly inhibited protein expression of MCM2 and cyclin-D1, but significantly increased expression of p21, compared with the negative-shRNA group (all P<0.01; Fig. 2D and E). No significant difference was identified in the cell cycle-related protein expression between the nontransfection group and the negative-shRNA group (all P>0.05).

### Effects of Notch2 knockdown on tumor growth and cumulative survival rate in nude mouse xenograft tumor models

**Figure 4:**
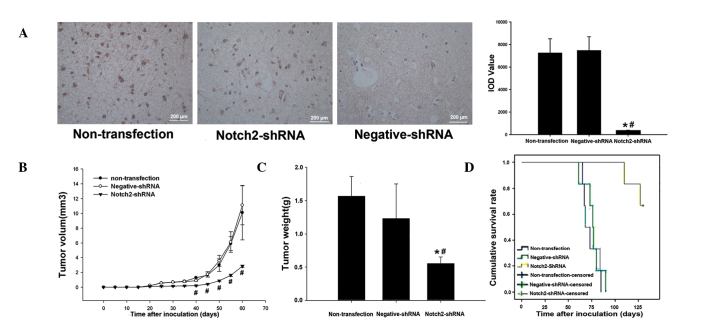
Effects of Notch2 knockdown on tumor growth and cumulative survival rate in nude mouse xenograft tumor models. (A) Notch2 protein expression in tumor tissues was determined by IHC on day 40 after inoculation (magnification, ×200). IHC quantitative analysis was shown as IOD (n=4). (B) Tumor volume. (C) Tumor weight on day 50 after inoculation (n=4). (D) Kaplan-Meier curves were plotted to determine cumulative survival rate and it was observed that the cumulative survival rate of rats in the Notch2-shRNA group (yellow curve) was higher than that in the negative-shRNA group (green curve, P=0.01). Data are expressed as the mean ± standard deviation. ^*^P<0.05, compared with the nontransfection group; ^#^P<0.05, compared with the negative-shRNA group. IHC, immunohistochemistry; shRNA, short hairpin RNA; IOD, integrated optical density.

IHC analysis showed relatively high protein expression of Notch2 in the tumor tissues inoculated with naive U87 cells or U87 cells stably transfected with negative-shRNA (yellow-brown to brown color), but almost no Notch2 protein expression was observed in the tumor tissues inoculated with U87 cells stably transfected with Notch2-shRNA on day 40 after inoculation (Fig. 4A). This was a statistically significant reduction (both P<0.05; Fig. 4A).

The tumor growth curve showed that tumor volume was significantly lower in the Notch2-shRNA group than that in the nontransfection and negative-shRNA groups 40 days after inoculation (P<0.05; Fig. 4B). On day 50 after inoculation, tumor weight in the Notch2-shRNA group was significantly lower than that in the nontransfection and negative-shRNA groups (0.55±0.1 vs. 1.57±0.29 and 1.23±0.52g, respectively; P<0.01; Fig. 4C).

The effect of Notch2 silencing on the cumulative survival rate in nude mouse xenograft tumor models is shown in Fig. 4D. In the nontransfection and negative-shRNA groups, tumor growth was rapid resulting in death on day 61–90 after inoculation. The survival time of mice in these groups was 73.7±3.6 days in the nontransfection group and 76.2±3.9 days in the negative-shRNA group. By contrast, in the Notch2-shRNA group, on day 130 after inoculation, four mice were alive and the survival time of the mice was 126.2±3.0 days. The cumulative survival rate was significantly longer in the Notch2-shRNA group compared with the negative-shRNA group (log-rank test, P=0.01). The cumulative survival rate was not identified to be significantly different between the nontransfection group and the negative-shRNA group (log-rank test, P=0.59).

## Discussion

Currently, the treatment of glioma, one of the most common malignant tumors of the central nervous system, consists of surgery, radiation and chemotherapy (1–5). Difficulty in completely removing tumors and the recurrence of cancer after treatment remains a significant obstacle to long-term survival. Thus it is crucial to develop novel therapies for the treatment of glioma, such as molecular cancer therapy. As it has been previously reported that the Notch signaling pathway is important for the initiation and development of tumors (14–21), it was investigated in U87 primary human glioma cells in the current study.

In the present study, substantial levels of Notch2 mRNA and Notch2 protein expression were detected in U87 human brain glioma cells. These results are in agreement with those reported by Chen *et al* (10,32), Reichrath *et al* (33) and Sivasankaran *et al* (30). Notably, Chen *et al* (10,32) and Reichrath *et al* (33) reported that Notch2 expression varied in different glioma cells, for example astrocytoma and medulloblastoma cells. This indicates that Notch2 may act as an oncogene or tumor suppressor protein, depending on the type of glioma (34–36).

In the current study, the effect of silencing Notch2 in cell proliferation was investigated using the MTT method. It was found that the Notch2 receptor was closely correlated with the level of proliferation of U87 glioma cells. Cell proliferation was significantly lower in the Notch2-shRNA group compared with the nontransfection and negative-shRNA groups. Cell cycle, determined by flow cytometry, showed that the proportion of cells in S phase was lower, whilst the proportion of cells in G_1_ phase was higher, in the Notch2-shRNA group compared with the nontransfection and negative-shRNA groups. After Notch2-shRNA cells were transplanted into nude mice, tumor growth was significantly suppressed, the number of tumors decreased and survival time increased. Similarly, Chen *et al* (32) reported that downregulation of Notch2 inhibited proliferation of U87 glioma cells *in vitro*. Jin *et al* (37) also showed that inhibiting the Notch signaling pathway with MRK003 can inhibit proliferation of U251 and U87 cells *in vitro.* In addition, it has been reported that suppressing the expression of Notch1 and Notch2 slows glioma cell proliferation *in vitro* (10). Here, Chen *et al*, showed that suppressing Notch2 expression was more effective in decreasing the rate of cell proliferation than the suppression of Notch1. By contrast, several studies have shown that Notch2 inhibits tumor cell growth by antagonizing Notch1 and that upregulation of Notch2 in U251 cells can suppress glioma cell proliferation (31,32,35,36). Consequently, it was hypothesized that Notch2 may have a dual effect on the proliferation of glioma cells. Several studies have investigated the effect of Notch2 on cell proliferation *in vitro*, but not *in vivo* (10,32,37). The current study confirmed the inhibition of glioma cell proliferation by downregulation of Notch2 *in vitro* and *in vivo*, thus providing valuable information for future studies on the role of Notch signaling in glioma.

The downstream Notch signaling pathways remain to be fully understood. Studies have shown that Notch signaling can regulate cell cycle progression and subsequent cell proliferation by multiple pathways (15,21–27,30,37). In the present study, it was found that Notch2 signaling in U87 human glioma cells increased the proportion of cells in the S phase and upregulated MCM2 and cyclin D1 protein expression levels; however, p21 protein expression was downregulated. This is consistent with previous studies that have shown that expression of downstream proteins of the Notch signaling pathway varies in different carcinoma cells. In small cell lung cancer cells, Notch signaling can increase p21 and p27 expression and hence induce cell cycle arrest (14). In mouse keratinocytes, Notch signaling inhibited cell cycle progression by increasing CSL-dependent p21 expression (38). A recent study has reported that Notch signaling induces cell cycle arrest by downregulating MCM2 and MCM6 protein expression (39). In the current study downregulation of Notch2 inhibited U87 cell proliferation by altering cell cycle-related protein expression and thereby regulating cell cycle progression.

The Notch signaling pathway is important for the initiation and development of tumors. In particular, the Notch2 receptor appears to be vital in the regulation of gliomblastoma cell proliferation (35,36). The present study indicates that downregulation of Notch2 mRNA and protein expression suppresses U87 human glioma cell proliferation *in vitro* and *in vivo,* and induces cell cycle arrest at the G_0_/G_1_ phase by upregulation of p21 expression, and downregulation of MCM2 and cyclin-D1 expression and cell apoptosis. The results of the present study indicate that the Notch2 signaling pathway is important in U87 human glioma cell proliferation. However, the molecular mechanisms underlying Notch2 regulation of glioma cell proliferation require further investigation.
